# Observations of Shear Stress Effects on Staphylococcus aureus Biofilm Formation

**DOI:** 10.1128/mSphere.00372-19

**Published:** 2019-07-17

**Authors:** Erica Sherman, Kenneth Bayles, Derek Moormeier, Jennifer Endres, Timothy Wei

**Affiliations:** a Department of Mechanical & Materials Engineering, University of Nebraska—Lincoln, Lincoln, Nebraska, USA; b Department of Pathology & Microbiology, University of Nebraska Medical Center, Omaha, Nebraska, USA; University of Iowa

**Keywords:** *Staphylococcus aureus*, biofilms, microchannel flow, shear stress, tower formation

## Abstract

Staphylococcus aureus bacteria form biofilms and distinctive microcolony or “tower” structures that facilitate their ability to tolerate antibiotic treatment and to spread within the human body. The formation of microcolonies, which break off, get carried downstream, and serve to initiate biofilms in other parts of the body, is of particular interest here. It is known that flow conditions play a role in the development, dispersion, and propagation of biofilms in general. The influence of flow on microcolony formation and, ultimately, what factors lead to microcolony development are, however, not well understood. The hypothesis being examined is that microcolony structures form within a specific range of levels of shear stress. In this study, laminar shear flow over a range of 0.15 to 1.5 dynes/cm^2^ was examined. It was found that microcolony structures form in a narrow range of shear stresses around 0.6 dynes/cm^2^. Further, measurements of cell density as a function of space and time showed that shear dependence can be observed hours before microcolonies form. This is significant because, among other physiologic flows, this is the same shear stress found in large veins in the human vasculature, which, along with catheters of similar diameters and flow rates, may therefore play a critical role in biofilm development and subsequent spreading of infections throughout the body.

**IMPORTANCE** It is well known that flow plays an important role in the formation, transportation, and dispersion of Staphylococcus aureus biofilms. What was heretofore not known was that the formation of tower structures in these biofilms is strongly shear stress dependent; there is, in fact, a narrow range of shear stresses in which the phenomenon occurs. This work quantifies the observed shear dependence in terms of cell growth, distribution, and fluid mechanics. It represents an important first step in opening up a line of questioning as to the interaction of fluid forces and their influence on the dynamics of tower formation, break-off, and transportation in biofilms by identifying the parameter space in which this phenomenon occurs. We have also introduced state-of-the-art flow measurement techniques to address this problem.

## Abstract (precis)

It is well known that flow plays an important role in the formation, transportation, and dispersion of Staphylococcus aureus biofilms. What was heretofore not known was that the formation of tower structures in these biofilms is strongly shear stress dependent; there is, in fact, a narrow range of shear stresses in which the phenomenon occurs. This work quantifies the observed shear dependence in terms of cell growth, distribution, and fluid mechanics. It represents an important first step in opening up a line of questioning as to the interaction of fluid forces and their influence on the dynamics of tower formation, break-off, and transportation in biofilms by identifying the parameter space in which this phenomenon occurs. We have also introduced state-of-the-art flow measurement techniques to address this problem.

## INTRODUCTION

Staphylococcal bacteria are recognized as the most frequent cause of biofilm-associated infections. This is primarily the case for lower respiratory tract infections and surgical site infections and secondarily for nosocomial bacteremia, pneumonia, and cardiovascular infections (1). Of particular interest in this study is the species Staphylococcus aureus, a nonmotile spherical bacterium with a diameter of ∼1 μm. Roughly 40% of the members of the general population are colonized with S. aureus. These individuals carry an increased risk for infection associated with surgery, dialysis, or intravascular device implants (2). Individuals who are colonized with S. aureus and who suffer from chronic diseases such as cystic fibrosis (3) are also at risk. The lethal reputation of S. aureus results from its ubiquity coupled with its ability to form biofilms and produce a variety of virulence factors.

Bacterial biofilm development varies with environment and species (4). S. aureus biofilm formation involves attachment, multiplication, exodus, maturation, and dispersal (5). The maturation stage is characterized by the emergence of “microcolonies,” or “tower” structures, arising from a basal layer of seemingly quiescent cells. Recent studies (6–8) have shown that different microcolonies exhibit heterogeneity in their growth and gene expression patterns, suggesting they might possess distinct functions within the biofilm population. For example, differences in metabolic activity associated with different microcolonies may result in mutagenic hot spots that promote genetic diversification (9). Another function might be to promote the dissemination of biofilm cells to distal sites. Indeed, previous studies (10–12) have shown that S. aureus biofilm formation is associated with the generation of so-called “rolling emboli,” which would presumably allow the cells associated with these structures to remain protected in a biofilm state while they transit to a new site.

Although formation of microcolonies is readily observed, little is known about what induces their formation. The effect of flow have been studied but not in the context of tower formation (10, 13–16). In the current study, the problem of identifying and understanding flow conditions under which S. aureus forms distinct tower structures was examined. Specifically, we tested the hypothesis that microcolony development is affected by fluid shear stresses. The results indicated that there is a distinct range of fluid shear stresses in which microcolonies form. Interestingly, the range of stresses around the level at which microcolonies formed (0.6 dynes/cm^2^) was similar to the shear stress found in large veins in the human vasculature, suggesting that this characteristic of S. aureus biofilm development can be optimized to promote dissemination within a mammalian host.

This investigation builds from an earlier study (5) in which S. aureus cells were cultured in a glass microchannel and subjected to flow conditions. It was observed that over the first ∼6 h, the cells appeared to multiply homogeneously and fill the microchannel floor. After that, there was an exodus event characterized by a sudden release of cells from the floor. From that point on, the biofilms developed microcolony structures that appeared as dark clumps of cells. In some cases, microcolonies would grow to the point of entirely blocking the channel. In other instances, the microcolonies would detach and be carried downstream by the flow.

**FIG 1:**
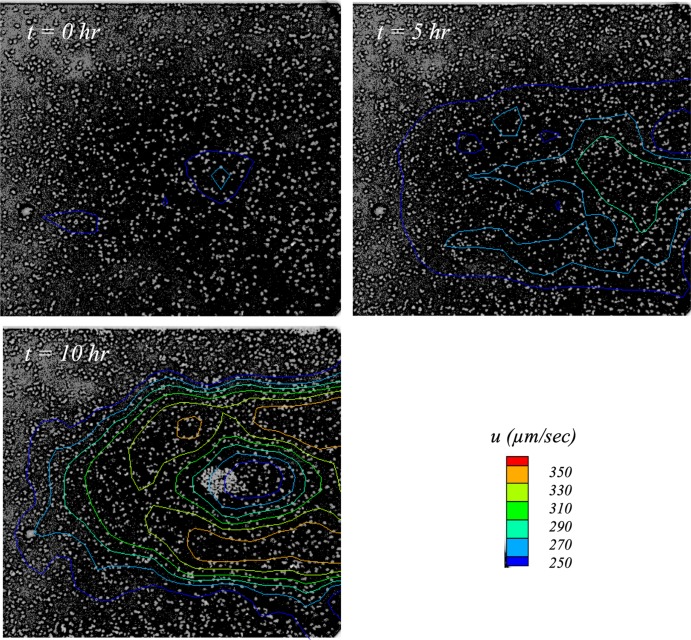
Contours of constant streamwise velocity overlaid on inverted (negative) images of S. aureus biofilm development in a preliminary experiment (17). Cells were subjected to a steady laminar flow with an applied shear stress of 0.6 dynes/cm^2^; flow is left to right. Observe the region of accelerated flow in the *t* = 5 h image (green contour) 5 h before the tower was first observed.

An additional driver for this investigation arose from a preliminary set of spatially and temporally resolved flow measurements of a tower formation event (17). The key finding from that experiment is shown in Fig. 1, in which of constant streamwise velocity measured ∼5 μm from the microchannel wall (using microparticle image velocimetry [μPIV]) are overlaid on microscope images of S. aureus. Note that the image intensities are inverted such that cells appear as bright circles on a dark background. Three images are shown for measurements made at 5-h intervals. Flow is left to right, and the field of view is ∼350 μm by ∼350 μm. The color legend indicates the velocity contour levels in micrometers per second.

The image at time (*t*) 0 h corresponds to initiation of flow. It simply shows that flow was uniform and that the cells appear to be randomly distributed across the field of view. In the third image (*t* = 10 h), the start of a tower is clearly visible to the right of center. Observe the isovelocity contours showing flow diverting around the forming tower.

The fascinating feature, however, is the middle image, corresponding to *t* = 5 h. Observe that the cells were still randomly distributed. However, the velocity contours indicate that flow appears to be diverging around the location where the tower would form 5 h later. It was determined in retrospect that cells in this particular experiment may have suffered from phototoxic effects that would negate any conclusions regarding normal biofilm growth. The data, however, fueled the hypothesis that flow itself plays an integral role in determining whether and where towers form.

The initial challenge in addressing this hypothesis is that of framing the parameter space in which the tower formation phenomenon occurs. Specifically, if flow does indeed affect tower formation, then there should be a well-defined set of flow conditions under which towers are most likely to form. Once that set of conditions is determined, subsequent research could be focused on understanding why and how those flow conditions affect tower development. While there is evidence (5, 18) that a shear stress of 0.6 dynes/cm^2^ is significant, this has not yet been quantitatively proven. The objective of this study, then, is to examine whether there is a preferred range of flow shear stresses for tower formation. Finally, it should be noted that because of the our previous work on the S. aureus strain, this particular study started at that point; examination of additional strains will be the focus of future work.

## RESULTS AND DISCUSSION

The goal of this investigation was to employ cell counting methodologies and flow measurement techniques to test the hypotheses that flow plays a decisive role in tower formation over a range of shear stress levels. As noted earlier, Staphylococcus aureus was used in this study because prior work from this team (5, 18) qualitatively indicated a biofilm phenotype dependence on shear for this strain. Experiments were conducted at six different applied shear stress levels from 0.15 dynes/cm^2^ to 0.9 dynes/cm^2^ in increments of 0.15 dynes/cm^2^. A seventh shear level, 1.5 dynes/cm^2^, was also tested to cover a full order of magnitude. The range was selected based on the empirical observation (18) that tower formation seemed most likely to occur around 0.6 dynes/cm^2^.

### Frequency and phenotype of tower formation.

The effect of fluid shear stress on formation of tower structures in wild-type UAMS-1 S. aureus is quantified in Table 1. Numbers of channels studied at each shear level and channels with observed towers are tabulated. It can clearly be seen that the applied shear stress of 0.6 dynes/cm^2^ had the highest occurance of tower formation at 0.33. Note that, since the microscope field of view was only in planes parallel to the microchannel floor on which the towers grew, it was not possible to ascertain the vertical growth of the towers.

**TABLE 1:** Tower-forming frequency as a function of applied shear stress for the seven applied shear stress levels^*a*^

Applied shear stress (dynes/cm^2^)	No. of channels tested	No. of channels with towers	Fraction of channels with towers	*P* values without Benjamini Hochberg adjustment	*P* values with Benjamini Hochberg adjustment
0.15	31	2	0.06	0.039	0.078
0.30	22	0	0.00	0.005	0.014
0.45	35	5	0.14	0.154	0.231
0.60	18	6	0.33		
0.75	12	1	0.08	0.193	0.232
0.90	18	2	0.11	0.228	0.229
1.50	24	0	0.00	0.004	0.014

a The *P* value from the Fisher exact test was found to be 0.001, implying that statistically significant differences exist in the frequency of observing towers as a function of applied shear stress. Comparisons of the six other shear stress levels against the level of 0.6 dynes/cm^2^ with and without the Benjamini Hochberg adjustment are shown.

Since all but two applied shear stress cases had fewer than five channels in which towers developed, the statistical significance of these findings was assessed using the Fisher exact test. The Benjamini-Hochberg method was then employed to determine the false-discovery rate for multiple comparisons. The *P* value from the Fisher exact test was found to be 0.001, implying that statistically significant differences existed in the frequency of observations of towers as a function of applied shear stress. Comparing the six other shear stress levels against the level of 0.6 dynes/cm^2^, the *P* values determined without the Benjamini Hochberg method were 0.039, 0.005, 0.154, 0.193, 0.228, and 0.004 (for 0.15, 0.30, 0.45, 0.75, 0.90, and 1.5 dynes/cm^2^, respectively). The corresponding *P* values after Benjamini Hochberg adjustments were 0.078, 0.014, 0.231, 0.232, 0.229, and 0.014.

This analysis clearly indicates a range of shear stress levels, 0.45 to 0.9 dynes/cm^2^, in which tower formation is more likely to occur. Further, Table 1 indicates a much narrower peak centered at 0.6 dynes/cm^2^, though the computed statistical significance of this is lower. Other results presented below, however, further distinguish 0.6 dynes/cm^2^ from the other applied shears.

**FIG 2:**
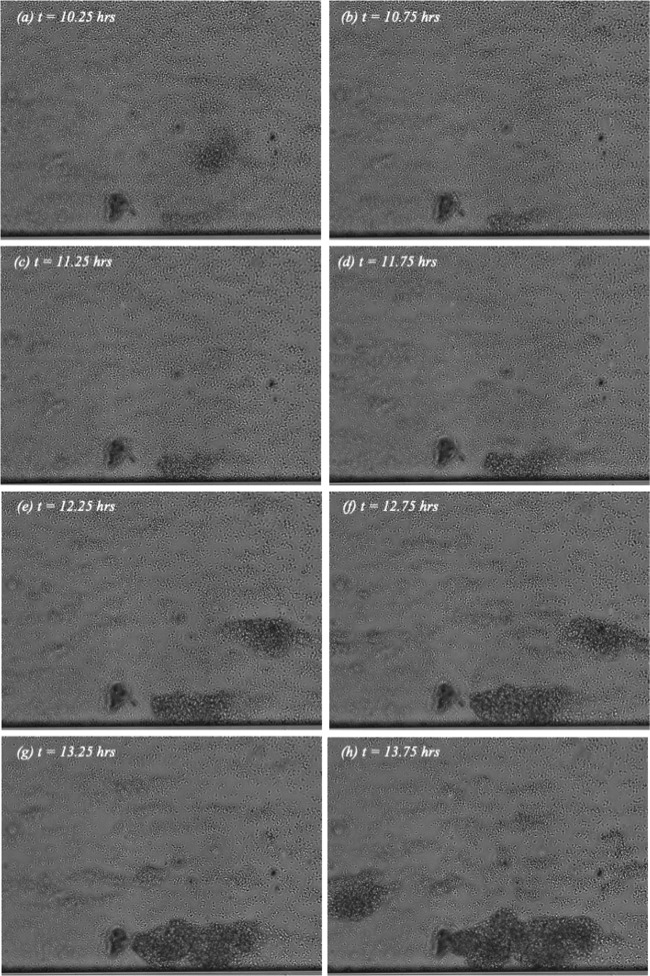
Sequence of eight video images showing tower formation at an applied shear stress of 0.6 dynes/cm^2^. Flow is left to right with *t *=* *0 corresponding to the start of experiment. The full 350-μm width of the channel spans each image from bottom to top.

Differences in tower phenotype for different shear levels appear in Fig. 2 (see also Fig. 4), where sequences of video images spaced at 30-min intervals (i.e., every sixth video image) are shown for shear stress levels of 0.6, 0.45, and 0.75 dynes/cm^2^. For each image, the time in minutes from the onset of flow is indicated. The viewing direction is through the microchannel bottom. Flow is left to right, with the full 350-μm microchannel width spanning each image bottom to top.

There are two tower phenotypes visible in the sequence of images corresponding to 0.6 dynes/cm^2^ in Fig. 2. The first is a large immobile tower that initiated and grew in place until it eventually completely blocked the channel (not shown). An example of this phenotype can be seen growing to the right of center along the bottom of the field of view.

The other phenotype is a large agglomeration of cells that does not anchor in place. Multiple examples can be seen in Fig. 2; they appear in one frame or two frames and then disappear. These are structures that likely formed upstream outside the field of view, broke loose from the microchannel floor, and then were carried downstream by the flow. Irrespective of phenotype, these towers can become quite large and dense.

**FIG 3:**
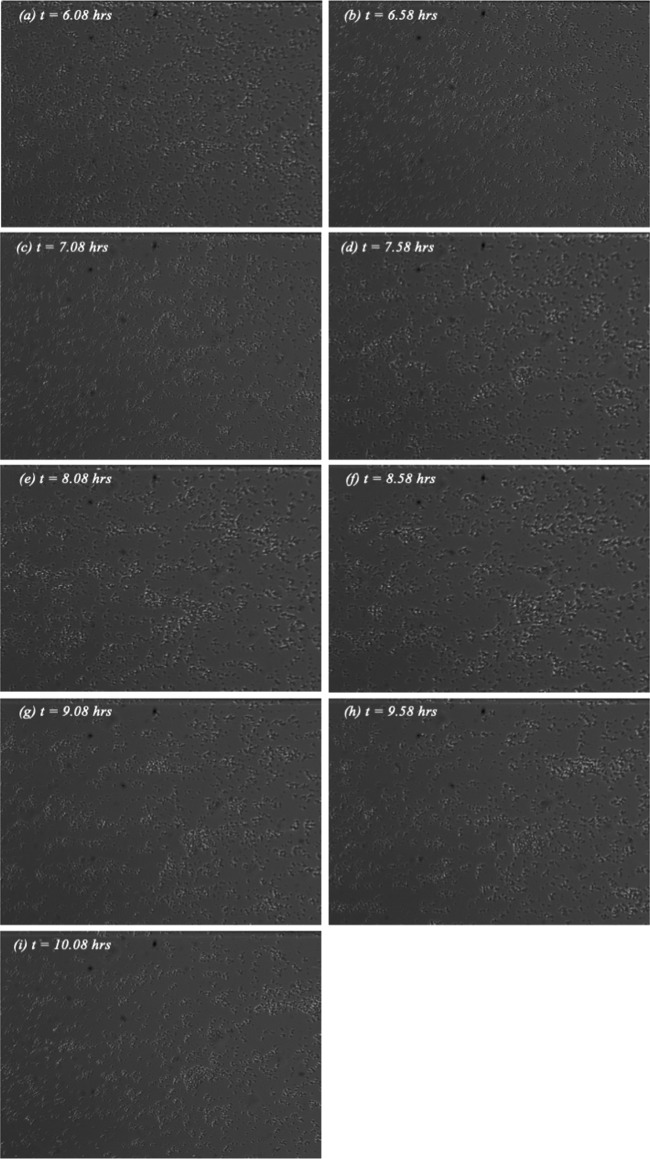
Sequence of nine video images showing tower formation at an applied shear stress of 0.45 dynes/cm^2^. The orientation, field of view, and reference time are identical to those in Fig. 2.

The towers observed at 0.45 dynes/cm^2^ (Fig. 3) are not similar to those generated at 0.6 dynes/cm^2^ shown in Fig. 2. The structures at this shear level were smaller and tended to shed cells and roll downstream. As seen in Fig. 3, cells appear to aggregate in smaller, less-dense structures that dynamically evolve and break free with time. Much lower cell density than that shown in Fig. 2 is also evident. This is discussed in greater detail in the following section.

**FIG 4:**
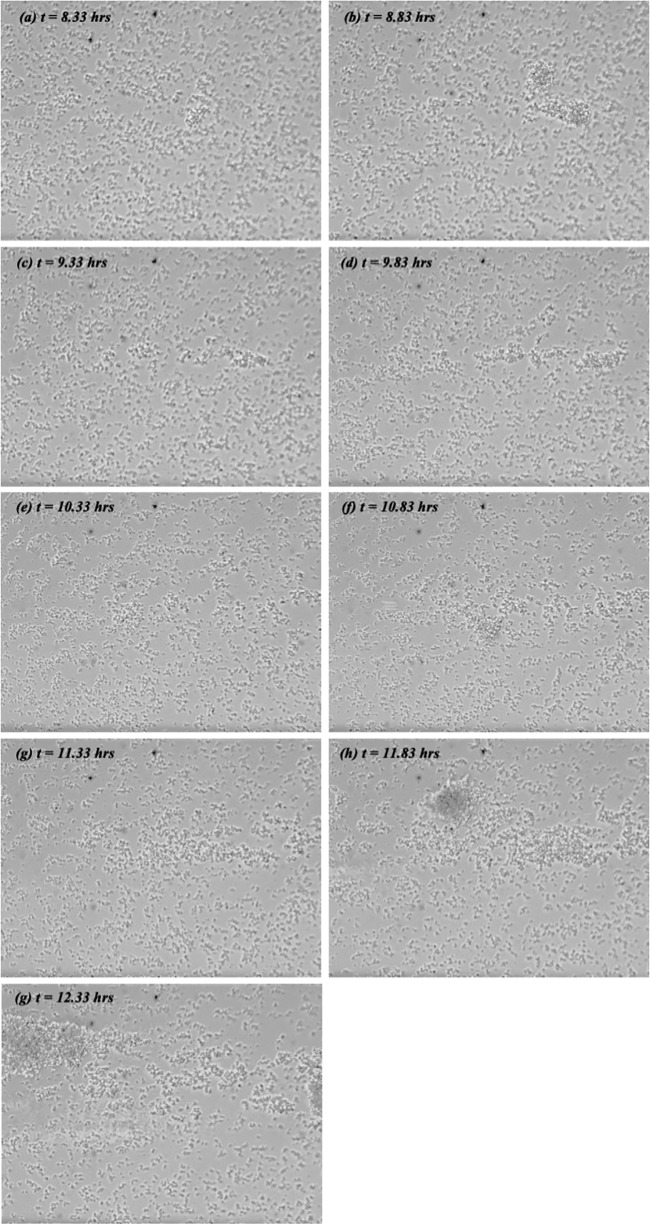
Sequence of nine video images showing tower formation at an applied shear stress of 0.75 dynes/cm^2^. The orientation, field of view, and reference time are identical to those in Fig. 2.

Finally, of the 12 channels examined at 0.75 dynes/cm^2^, only one tower was observed. Development of this tower is shown in Fig. 4. One can see that it formed above the microchannel midline though its shape, and its location appears to have varied somewhat with time but, overall, it appears to have been elongated in the flow direction. And, by and large, the tower shown in Fig. 4 appears less dense than the ones seen in Fig. 2, though differences in lighting make that interpretation somewhat uncertain. In general, however, it was observed that the towers forming at 0.6 dynes/cm^2^ were larger and more firmly affixed to the channel floor than the towers observed at other applied shears.

While understanding the exact reason for this apparent preference for 0.6 dynes/cm^2^ is beyond the scope of this study, it is worth referring back to the literature to highlight why flow would be important in microcolony formation and detachment. Specifically, since S. aureus does not employ swimming mechanisms such as flagella, its sensitivity to the flow environment may be essential to its dispersion strategy. In fact, studies in curved geometries where S. aureus has been shown to form biofilm streamers at corners seems to indicate that by forming streamers, S. aureus benefits from the mass transport of new cells to the streamer location, fortifying its porous structure and facilitating its growth to block channels (6). Generally, motility may enable a cell to acquire nutrients or to move toward more favorable conditions (14). In fact, high shear flows may prove challenging for swimming or motile bacteria to travel toward new areas or nutrients, as such conditions tend to promote surface attachment (19).

Perhaps more importantly, it appears that the shear stress level of 0.6 dynes/cm^2^ is clinically very significant. *In vivo* measurements of flow in arteries and veins (20, 21) indicate that the mean shear level in the larger veins and arteries is less than 1.0 dynes/cm^2^. In fact, magnetic resonance imaging (MRI) in a healthy vein (21) yielded wall shear stress levels of exactly 0.6 dynes/cm^2^. This is particularly important because venous flow is steady, unlike the pulsatile flow in arteries. As such, the conditions examined in the current study are more relevant to flow in veins. It would be expected that catheters with diameters and flow rates similar to those measured for veins would have wall shear stresses in a comparable range.

### Temporal variations in cell density.

**FIG 5:**
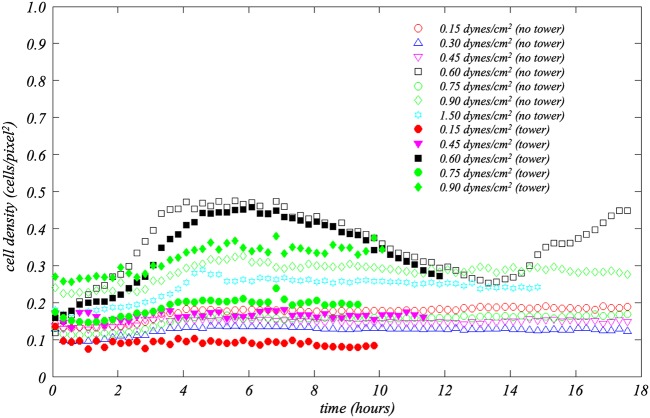
Average cell density (i.e., number of pixels occupied by cells divided by the total number of pixels in the field of view) versus time for the seven different applied shear stresses. Data for each shear are further segregated by whether or not a tower formed in the channel. Data corresponding to the numbers of channels for each case can be extracted from Table 1. Observe how distinctly different the 0.6 dynes/cm^2^ case is for both channels with and without towers relative to the other applied shears.

To quantitatively examine differences between flows with and without towers, temporal and spatial variations in cell density and flow were studied. As described under “Image processing and analysis” below, cell densities in regions of 8.4 μm by 8.4 μm (25 by 25 pixels) in the fields of view were computed. The average cell density in a channel at each time step was calculated on the basis of the 2,255 regions comprising the entire channel field of view. Data from matching cases, i.e., same shear stress with tower formation or same shear without towers, were then further determined by ensemble averaging. Data corresponding to average cell density as a function of time for the seven shear stresses examined appear in Fig. 5. Solid symbols indicate averages for tower cases, while open symbols were used for nontower cases.

It can be observed in Fig. 5 that the cell density values for the majority of shear stress levels are <0.2, irrespective of whether towers formed or not. The notable exception is 0.6 dynes/cm^2^ with and without towers. The observation should be made that the 0.9-dynes/cm^2^ cases, i.e., with and without towers, and the 1.5-dynes/cm^2^ case without towers have maximum average cell density values between 0.25 and 0.37. It is also interesting that the mean cell density in the 0.9-dynes/cm^2^ cases appears to have been higher than in any other case at the start of the experiments; this is a point for futher investigation. The key point here, however, is that the cell densities for the 0.6-dynes/cm^2^ cases are dramatically higher than all other shear levels, both with and without towers. Along with the video records, this further highlights the shear level of 0.6 dynes/cm^2^ as biologically significant.

Focusing on the two 0.6-dynes/cm^2^ curves, cell multiplication in the first 4 h is clearly visible. Indeed, this growth rate is dramatically higher than that seen with any other applied shear. Interestingly, the cell growth rate was lower in the channels that ultimately developed towers than in those in which towers did not form. This is a distinction that is actually noticable in the data corresponding to the period within the first hour of the experiment and over 10 h before towers develop. (Data for the channels where towers formed end at the time point at which the towers first appeared.)

The exodus phenomenon, i.e., the release of cells attached to the microchannel floor, is visible beginning at approximately *t* = 7 h as a decrease in cell density. It turns out that whereas the onset of exodus is visually distinct in the videos, cell density continued to decrease in the 0.6-dynes/cm^2^ channels for several hours. In fact, for the period of 4 h < *t *<* *12 h when towers formed, the two 0.6-dynes/cm^2^ curves with and without tower formation are indistinguishable.

### Spatiotemporal variations in cell density.

Having divided the camera field of view into regions of 8.4 μm by 8.4 μm (25 by 25 pixels), it was possible to also quantify the spatial distribution of particles at each time step. This is done by computing probabilility density functions (pdf) of the cell densities. That is, defining ρ*_i_*_,_*_j_*(*t*) as the cell density in the (*i*, *j*) region of the field of view at time *t*, the probability that the cell density value in that region would fall within some interval, **ρ** ≤ ρ*_i_*_,_*_j_*(*t*) ≤ **ρ + *Δ*ρ**, corresponds to the number of regions with density values in that interval divided by the total number of regions. The pdf at time *t* then represents the composite of probabilities for all intervals 0 ≤ **ρ** ≤ 1. By generating pdfs at regular time intervals across the biofilm development, one can look for additional differences between the channels that formed towers and those that did not.

**FIG 6:**
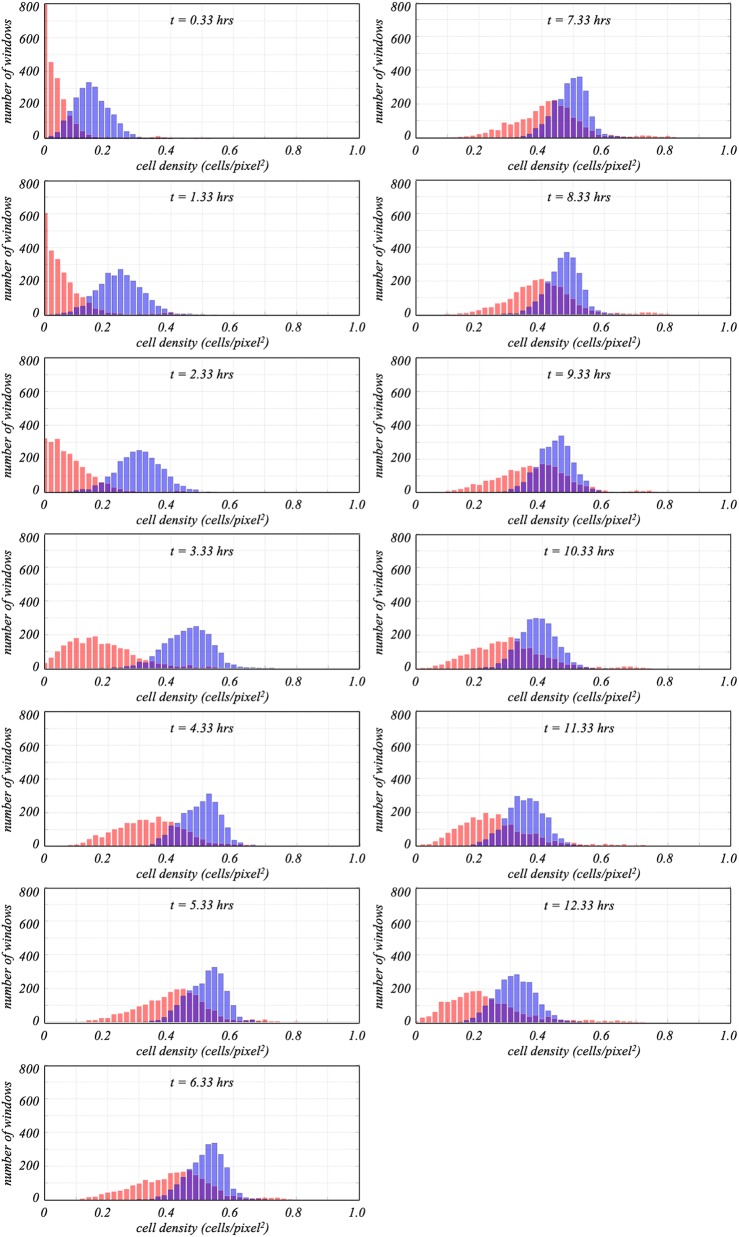
Cell density pdf graphs (representing the number of 25-by-25-pixel windows with a certain cell density) for the channels corresponding to a shear stress of 0.6 dynes/cm^2^. Data for channels with towers are shown in red. Nontower data are blue. As indicated in Table 1, there were 6 channels in which towers were observed and 12 without. Panels *a* to *m* compare pdfs determined at 1-h intervals beginning 20 min after flow was initiated. On average, towers formed <12 h after the start of the experiments.

The evolution of cell density pdfs for the 0.6-dynes/cm^2^ channels appears in Fig. 6. Ensemble-averaged pdfs for channels with (shown in red) and without (blue) towers are shown at 1-h intervals beginning 20 min after shear was applied. Key features are the location, symmetry, and width of the individual pdfs. The location of each pdf represents the mean cell density. Thus, if cell density increases, the centroid shifts to the right. Observe that this occurs for the channels both with and without towers. The pdfs for the channels with towers, however, are distinctly to the left of the pdfs for the channels without towers, particularly at earlier times (Fig. 6a to c). This is consistent with the data in Fig. 5 showing that, overall, the cell density for the 0.6-dynes/cm^2^ channels with towers is lower than for channels without, particularly during the multiplication phase.

Symmetry, in turn, indicates the homogeneity of the pdf. A tail extending to the right in the pdf indicates that cells are concentrated in small regions in the field of view, while a tail to the left indicates patches where there are fewer cells. Comparing pdfs of channels with and without towers in Fig. 6 reveals that pdfs of channels without towers are generally symmetric whereas pdfs of channels with towers start off highly asymmetric, with tails extending to the right. This indicates that even at the earliest stages of the experiments, the cells in the channels that ultimately formed towers tended to culture in clumps rather than distributing uniformly across the channel.

Finally, while the widths of the pdfs for channels without towers are roughly constant for the duration of the runs, pdfs for channels with towers broaden with time. In Fig. 6a, at ∼20 min after flow was started, the pdf for the channels that ultimately formed towers is highly asymmetric, with a maximum at a value of 0.0, and, except for a very small secondary peak around 0.35, is confined to density values of <0.2. For each subsequent hour, those pdfs widen and move to the right. The secondary peaks at the extreme right of those pdfs exist for all times analyzed and indicate a small number of regions in which the cell density is significantly higher than in the rest of the field of view. Hours before towers form, then, the spatial distribution of cells for the channels that formed towers was different from the distribution for those that did not.

### μPTV measurements.

There are clearly differences in cell density and distribution when towers do and do not form. These differences are most distinct for an applied shear of ∼0.6 dynes/cm^2^. The issue remains, however, of whether there are mechanics-based indicators, e.g., fluid velocity, that may serve as predictors for tower formation. To test this, microparticle tracking velocimetry (μPTV) studies were conducted.

It should be noted at the outset that a result as clean as that shown in Fig. 1 was never replicated. That is, there does not appear to be a simple, recognizable fluid velocity indicator that foreshadows the imminent appearance of a tower. However, through multiple experiments and measurement refinements performed over several years, a consistent observation was that the flow seen when towers form is more variable than when towers never form.

The most compact way of quantifying these differences was to calculate the spatial mean, *U*, and root mean square (RMS), *u*′, corresponding to variations of streamwise velocity with time. This is analogous to the cell density statistics described earlier in this section. The key difference is that, for this analysis, streamwise velocity instead of cell density was the dependent variable of interest.

Specifically, for each of the six channels in which towers were observed, rectangular regions were defined surrounding the location where each tower ultimately formed. This was done retrospectively by looking at the end of the video record and identifying the size and location of the tower(s). For an example, the reader is referred to the tower along the bottom of the image in Fig. 2h. Note that the size of the bounded region depended, of course, on the size of the tower that formed.

At each time step, beginning after exodus, the mean and RMS values corresponding to every streamwise velocity value in each region were computed; these values represent the spatial mean and RMS for each region at that time. This was done for each time step until a tower formed. For channels where no towers formed, equivalently sized regions were defined and analogous time histories of *U* and *u*′ were computed.

To remove channel-to-channel variability in the bulk flow, all values of *U* were made nondimensional by application of the spatial mean velocity at the first time step after exodus for the corresponding channel, *U_o_*. In that way, the first nondimensional mean velocity value for every channel was unity, i.e., *U*/*U_0_* = 1. The RMS variations in velocity, *u*′, were made nondimensional by application of the spatial mean velocity at the corresponding time step, *U*. At each time step, then, *U*/*U_o_* and *u*′/*U* for the 12 0.6-dynes/cm^2^ channels without towers were determined by ensemble averaging. The same was done for the six channels in which towers formed. The full data set is presented in the work by Sherman (22).

It was observed that for the channels in which no towers formed, *U*/*U_o_* increased ∼5% over the first 3 h after exodus and another ∼10% in the following 1.5 h. In contrast, the *U*/*U_o_* value decreased by ∼10% in the regions where towers did form in the first 3 h after exodus. After the towers began forming, *U*/*U_o_* decreased precipitously as flow diverted around the forming towers.

There were also differences in *u*′/*U* values between the tower and nontower cases. For the first 3 h after exodus, the nondimensional RMS variations in streamwise velocity, *u*′/*U*, for the channels without towers is ∼0.00025%. In contrast, for the channels with towers, *u*′/*U* increases from 0.0013 to 0.0015. Even though the flow is nominally laminar in both cases, the streamwise variations in the channels with towers are roughly six times greater than those in the channels without towers. Once towers form, the mean speed decreases and the flow diverts around the towers, resulting in a dramatic increase in *u*′/*U* to 0.0087.

### Conclusions.

A detailed study of tower formation in S. aureus biofilms subjected to laminar shear flow was performed. Video records of flow in microchannels were made over a range of 1 order of magnitude of applied shear stress from 0.15 dynes/cm^2^ to 1.5 dynes/cm^2^. Data about cell density and spatial distribution of cells as a function of time were gathered along with spatially resolved μPTV measurements of the flow field at successive times in the biofilm development. Analysis of these data yielded the following observations. There is a statistically significant range of shear stresses, i.e., between 0.45 dynes/cm^2^ and 0.75 dynes/cm^2^, where tower formation is more likely to occur. The highest probability of tower formation occurs at 0.6 dynes/cm^2^. Time histories of mean cell density and visual records of biofilms across the entire range of shear stresses studies further indicate unique behaviors of biofilms at 0.6 dynes/cm^2^. *A posteriori* analysis of the data indicated that there are differences between flows with towers and flows without that appear hours before tower formation, in some cases even before exodus. And the value of 0.6 dynes/cm^2^ corresponds to wall shear stress levels in larger human blood vessels and catheters of similar diameters and flow rates.

In conclusion, then, specific to the two hypotheses tested, it does indeed appear (i) that there is a distinct range of fluid shear stresses in which towers occur and (ii) that there are indicators of tower formation that manifest hours before towers form. At this juncture, the exact nature of the coupling between fluid dynamics and tower formation is not clear. There is also a need to expand this study to examine other bacterial strains. However, the key finding from this study is that there is indeed a connection of potentially very significant clinical importance that warrants further investigation.

## MATERIALS AND METHODS

### Microchannel and imaging system.

The S. aureus strains used in this study were derived from osteomyelitis isolate UAMS-1. All experiments were initiated with fresh overnight cultures grown at 37°C in tryptic soy broth (TSB) with shaking at 250 rpm. A Bioflux 1000 microfluidic system, including a Nikon Ti-S inverted microscope, hardware controllers for pump and microscope stage manipulation, a vapor trap to reduce condensation, a pressure interface to connect the pump to the plates, and a heating plate, was used.

Experiments were conducted in polydimethylsiloxane (PDMS) microchannels (350 μm wide by 70 μm high by ∼4,000 μm long) with glass bottom walls. A pneumatic pump produced a steady laminar flow with wall shear stresses in the range 0 to 20 dynes/cm^2^. The microchannel plates, with 24 or 48 wells per plate, were mounted on a three-axis stage that could also be positioned to 0.1-μm accuracy. The stage included a heater that maintained a temperature of 37°C. For these studies, the *x*, *y*, and *z* axes were aligned with the flow, width, and height directions, respectively. Imaging was in *x-y* planes.

Two digital video cameras were used in this investigation. A Retiga EXi CCD camera with resolution of 1,392 by 1,040 pixels was used for imaging and counting cells attached to the floor of the microchannels. These images were used to compute statistical data about the biofilms such as cell density and distribution as a function of time.

A Phantom Miro M310 high-speed color camera was used to make μPTV flow measurements. This technique is described below. The camera has a thermoelectrically cooled complementary metal oxide semiconductor (CMOS) sensor with resolution of 1,024 by 768 pixels and can capture full frame video at over 3,000 frames/s.

The cameras were mounted to a Nikon Ti-S inverted microscope (part of the Bioflux system). A Nikon Plan Fluor ELWD Ph2 DM objective (40×/0.60 numerical aperture [NA]) was used with an LED microscopic lamp as the illumination source. The lamp was operated at 40 W to avoid disrupting the normal biological activity of the bacteria.

### Microparticle tracking velocimetry (μPTV).

Microparticle tracking velocimetry (μPTV) is a noninvasive flow measurement technique that involves tracking particle images in successive frames from a video record of flow seeded with small, neutrally buoyant particles. For large-scale (e.g., aerodynamics) experiments, a sheet of laser light is typically used to illuminate particles in a two-dimensional plane. Flow measurements with micrometer resolution (i.e., μPTV) are made using a camera mounted to a microscope such that the microscope objective’s focal plane defines the measurement plane. Video images are digitized, and a computer algorithm computes displacements of every particle in the field of view from one frame to the next. By noting the location of each particle and the time between successive video frames, this process yields a two-dimensional vector field in which flow velocity is measured at every particle location. Subpixel particle displacement resolution is achieved by computing the centroid of each particle image. Creating an ensemble of vector fields increases spatial resolution. Once the resolution is sufficiently high, a uniformly spaced vector field is created by interpolating the data from the ensemble of fields. Please see the work by Lambert (23) for further details.

In this study, individual (∼1-μm-diameter) S. aureus cells suspended in the flow served as μPTV seeding particles. This was done for two reasons. First, as cells were pumped into the microchannels and during subsequent multiplication stages, there would always be a number of cells that did not attach to the microchannel walls. As such, there were always enough suspended cells for accurate flow measurement, even early in the experiments when cell densities were low. These free-floating cells were nominally neutrally buoyant and were the right size for μPTV flow measurements. More importantly, it was observed that suspended S. aureus cells agglomerated around the standard seeding particles typically used for μPTV. As such, seeding particles became nucleation sites for biofilms and significantly altered the biofilm formation.

### S. aureus preparation.

In order to grow biofilms in the Bioflux system, microchannels were first primed for 5 min with 200 μl of TSB at 5.0 dynes/cm^2^. After priming, the TSB was replaced with 200 μl of fresh overnight cultures diluted to an optical density (OD) of 0.8. Channels were then seeded by pumping from the output wells to the input wells at 2.0 dynes/cm^2^ for 5 to 10 s. Cells were allowed to attach to the channel surfaces for 1 h at 37°C. At the start of each experiment, excess inocula were carefully aspirated off and 50% TSB was pumped through the microchannels at the desired flow rate, i.e., under shear stress conditions, for 16 h.

### Flow conditions and imaging.

Experiments were conducted at flow rates of 16, 32, 48, 64, 90, 106, and 160 μl/h, corresponding to wall shear stress levels of 0.15, 0.3, 0.45, 0.6, 0.75, 0.9, and 1.5 dynes/cm^2^, respectively. The working fluid was 50% TSB. For reference, 0.6 dynes/cm^2^ (64 μl/h) and 1.5 dynes/cm^2^ (160 μl/h) corresponded to microchannel Reynolds numbers, based on mean flow speed and channel height, of 0.001 and 0.0025, respectively; the flow was laminar for all cases studied.

As noted previously, two types of imaging were done in this investigation, one for cell counting and the other for μPTV flow measurements. For cell counting and for subsequent statistical analysis of cell counts, a Retiga EXi charge-coupled-device (CCD) camera was used to record images at 5-min intervals for approximately 16 h. By that time, experience showed that either at least one tower would form in the field of view or it was unlikely that one would be observed. With the Bioflux plates, multiple channels were run simultaneously. The microscope stage positioning software sequentially cycled through each microchannel such that one image per channel was recorded every 5 min. Every image in each channel was recorded at the same location ± 0.1 μm.

Images for μPTV experiments were recorded with a Phantom Miro M310 high-speed color camera. The time steps were every 45 min instead of every 5 min. This enabled capture of 4,000 images in each channel per time step at a capture rate of 50 pairs/s.

### Image processing and analysis.

Two different image processing techniques were used to identify S. aureus cells, one for determination of biofilm formation statistics and the other for μPTV measurements. In the former, the focus was on stationary cells attached to the microchannel floor. For the flow measurements, it was important to identify only the unattached cells being carried by the flow.

For biofilm cell density measurements, images were inverted and subsequently converted to binary data using a threshold appropriate to the specific data set. Averages of three consecutive images (captured every 5 min), centered on the middle image, were generated to identify nonmoving cells. In this manner, the time between cell density measurements was maintained at 15 min.

To calculate spatial variation of cell density every 15 min, the averaged image was divided into regions of 25 by 25 pixels, i.e., ∼8.4 μm by 8.4 μm. Cell density in each region was defined as the number of bright pixels divided by the 625 pixels in the region. The cell density of the entire field of view was simply the average of the cell densities in regions of 25 by 25 pixels in each image.

For μPTV, color images were converted to grayscale and thresholded to create binary images. For each time step, a background image was generated using 100 image pairs. This was subtracted from every image in the video sequence to produce images containing only moving particles. Finally, electronic noise was digitally filtered prior to using the μPTV algorithm.

### Data availability.

The results determined in this study derived from ∼50,000 video images totaling 50 GB of data. As described, image processing to identify, track, or count cells comprised the bulk of the data analysis. The video records can be made available by a request to the corresponding author.
